# Loss rate of net primary productivity under drought stress on the Yinshanbeilu of Inner Mongolia, China

**DOI:** 10.3389/fpls.2025.1438343

**Published:** 2025-02-19

**Authors:** Wang Sinan, Yingjie Wu, Wenjun Wang, Jianyin Guo, Mingyang Li

**Affiliations:** ^1^ Institute of Water Resources of Pastoral Area, Ministry of Water Resources, Hohhot, China; ^2^ Yinshanbeilu Grassland Eco-Hydrology National Observation and Research Station, China Institute of Water Resources and Hydropower Research, Beijing, China; ^3^ Shandong Provincial Key Laboratory of Water Resources and Environment, Water Resources Research Institute of Shandong Province, Jinan, China

**Keywords:** net primary productivity, drought, response, loss rate, CASA model

## Abstract

**Introduction:**

The increasing intensity and frequency of droughts seriously threaten the structure and function of terrestrial ecosystems. In order to ensure the normal play of ecosystem service function under future stress, the temporal and spatial characteristics of ecosystem productivity response to drought need to be explored.

**Methods:**

The net primary production (NPP) of vegetation in the Yinshanbeilu was calculated using the Carnegie-Ames-Stanford Approach (CASA) model, and subsequent study concentrated on the NPP’s geographical and temporal variable characteristics. By the calculation of the standard precipitation evapotranspiration index (SPEI), the study also sought to examine the relationship between drought and NPP at various time scales. Researchers also built drought loss rate curves based on various fertility stages using the vulnerability curve construction method.

**Results and discussion:**

Findings revealed that the SPEI had varying degrees of efficacy in capturing drought conditions at various time frames. Nonetheless, the SPEI’s spatial distribution, which shows a wet distribution in the east and an arid distribution in the west, exhibited identical characteristics for all scales and may be used to indicate drought. Significant interannual variation was seen in the NPP of the study area’s vegetation, which fluctuated in an upward direction from 2000 to 2020. 75.89%, 77.23%, 81.35%, and 83.56% of the area were found to have a positive correlation between the SPEI and vegetation NPP at various time scales, with 42.53%, 48.15%, 90.72%, and 92.75% of the area passing the significance test (*p* < 0.05), in that order. Their results showed that as the SPEI time scale was increased, the link between vegetation NPP and SPEI became stronger. The loss rate of vegetation NPP fluctuated and grew regularly with the expansion of drought degree, varying between 20-50%, according to drought loss rate curves created for each fertility period.

## 1 Introduction

It is widely acknowledged that drought is a serious natural disaster with huge global economic repercussions. In fact, drought-related economic losses account for a substantial portion of all natural disaster losses (Liu et al., 2020a). Additionally, the persistence of drought can lead to a range of cascading effects, including reduced soil water resources for vegetation growth, inhibited photosynthesis, and the induction of natural disasters such as dust storms and fires. These consequences may drastically reduce ecosystems’ capacity to serve as carbon sinks, which may eventually lower vegetation productivity (Nejadrekabi et al., 2022; Tian et al., 2019; Wang et al., 2022a). As a result, understanding the impacts of drought on vegetation presence is crucial for enhancing ecosystem stability (Ding et al., 2022; Geng et al., 2022; Shi et al., 2022; Soleimani-Motlagh et al., 2022). Furthermore, given the potential for increases in the intensity, frequency, and duration of drought, it is essential to explore strategies aimed at mitigating these negative effects and preserving the functional structure of terrestrial ecosystems.

Numerous researchers have made use of drought indices in order to accurately measure complex instances of drought. These indices, which include the Palmer Drought Index (PDSI), the Standardized Precipitation Index (SPI), and the Standardized Precipitation Evapotranspiration Index (SPEI), are designed to more accurately reflect the spatial and temporal characteristics of drought (Laimighofer and Laaha, 2022; Ramirez et al., 2022; Wahla et al., 2022; Xu et al., 2022). Several scientific investigations have indicated that vegetation responses to drought stress involve the closure or reduction in size of vegetation stomata, impacting CO_2_ uptake, and subsequently, vegetation photosynthesis (Gang et al., 2022; Wei et al., 2022a), These effects are particularly pronounced in areas characterized by arid and semi-arid weather, which feature more vulnerable terrestrial ecosystems (Geng et al., 2023). Specifically, a widespread drought incident markedly diminishes the net primary productivity of plant communities (Li et al., 2020a). Furthermore, it has been observed that vegetation NPP and SPEI are predominantly positively correlated, particularly in the 20-50°N latitude range, where vegetation is more sensitive to drought stress (Liu et al., 2021b). As droughts become more frequent and persistent, the loss in vegetation NPP amplifies significantly (Sun et al., 2016). The reaction of NPP to drought displays geographical variability and is subject to multiple uncertainties during analysis (Khatri-Chhetri et al., 2021). One illustration of this is the research of Kljun et al. (2007) on poplar forests in southern Canada, which revealed that severe drought events of extended duration and high intensity suppressed both vegetation respiration and photosynthesis to a similar degree, ultimately resulting in little alteration of vegetation NPP. However, under mild or moderate drought conditions, ecosystem photosynthesis remained largely unaffected while respiration was considerably reduced, and this led to the observed increase in vegetation NPP. Consequently, it is clear that while current studies tend to concur that drought-induced water stress typically reduces vegetation NPP via stomatal closure, there is considerable variability in the response to drought across different regions, biomes, and land use types, as well as in relation to ambient climate conditions, which can all influence vegetation response (Tong et al., 2023; Yuan et al., 2022; Zhao et al., 2019a). Currently, the majority of studies are concentrated on the decrease in NPP during drought episodes. However, an essential aspect regarding quantifying the susceptibility of vegetation NPP to drought has yet to be examined. Additionally, additional exploration into the temporal response of NPP to drought is necessary.

There is a fragile ecological zone in China that is arid and semi-arid, and the Yinshanbeilu region of Inner Mongolia is located within this zone. As a consequence of this, the amount of moisture that is accessible plays a significant part in determining the degree to which vegetation is able to prosper in this area (Wang et al., 2022b). A number of ecological problems, including ecological and vegetation degradation, as well as water shortages, have surfaced as a direct result of the exacerbation of climate change brought on by human activities in the region. The main objectives of this paper were to (1) reveal the spatial and temporal evolution of multi-scale drought in grasslands and to identify the years of drought occurrence; (2) estimation of NPP by CASA model to reveal the spatial and temporal variation pattern of regional NPP; and (3) construct a drought disaster loss rate curve for vegetation during the agricultural production period and quantitatively evaluate the effects of different drought levels on vegetation productivity. This offered a plausible method for promptly assessing the loss due to drought in vegetation. This research is an innovative attempt in the field of assessing the effects of drought on vegetation, and it offers important new theoretical insights.

## 2 Materials and methods

### 2.1 Study area

**Figure 1:**
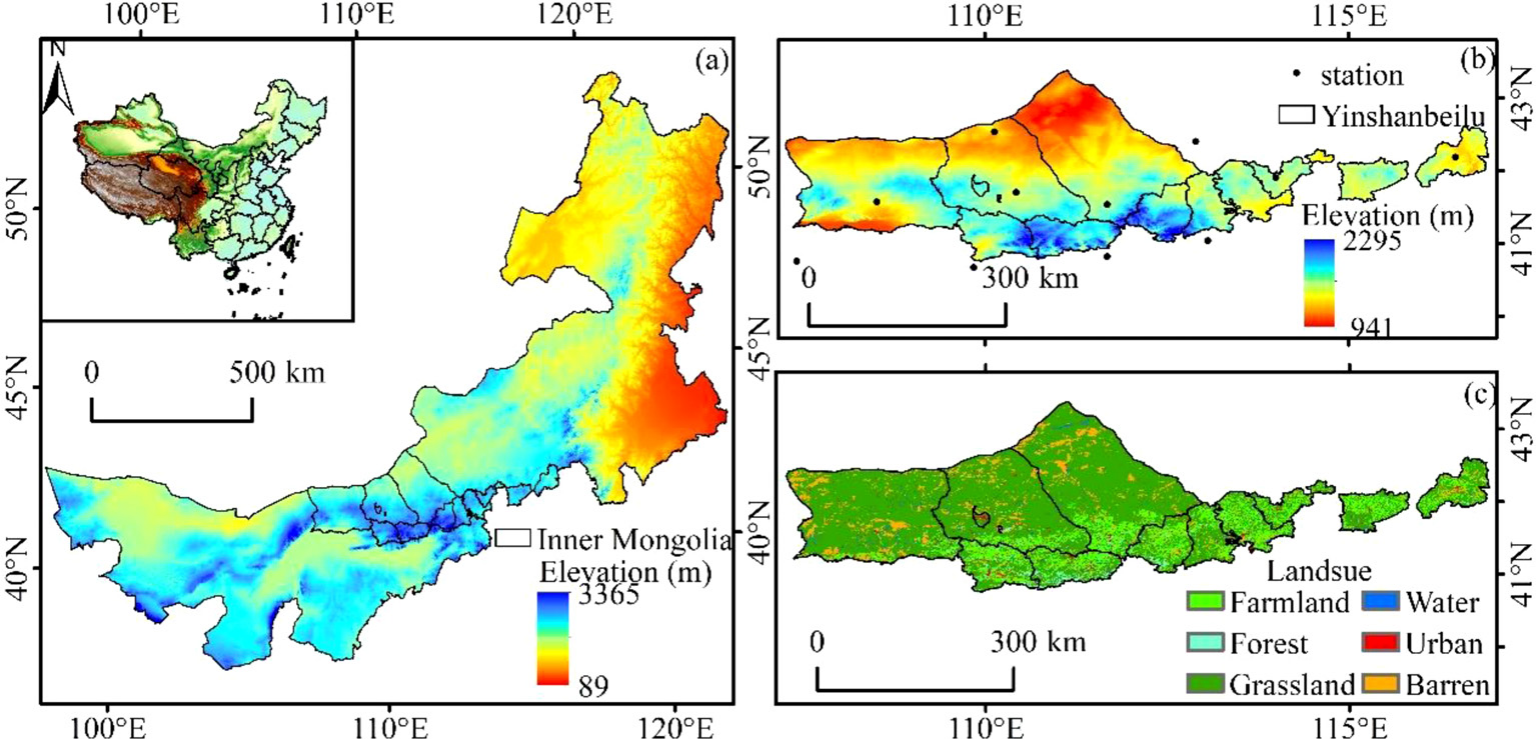
Location of the study area. **(A)** location relative to Inner Mongolia, **(B)** Elevation, meteorological stations, and **(C)** land cover.

Yinshanbeilu is a transitional agricultural and pastoral area situated between the Yinshan Mountains and the Mongolian Plateau (**Figure 1**). The coordinates are 107°17′~116°53′E and 40°43′~43°23′N, respectively. Twelve banners and counties make up the administrative jurisdiction, which covers a massive 97,250.5 km^2^. temperatures averaging 1.3–3.9°C, evaporation rates averaging 1,748–2,300 mm, precipitation averaging 200–400 mm, and frost-free periods typically ranging from 102–121 days. Soil wind erosion, desertification, soil erosion, land degradation, and other ecological and environmental problems are becoming increasingly serious as a result of land use changes and human activities, severely limiting the growth of local economies and societies (Li et al., 2023).

### 2.2 Data sources

#### 2.2.1 Remote sensing data

NDVI data using MOD13A2 (https://search.earthdata.nasa.gov/search), the time series of 2000-2020, the 16 days of synthetic products, and a spatial resolution of 1 km. MCD12Q1 yearly synthetic products from 2000 to 2020 were used to collect vegetation type data. Time series from 2000 to 2020 utilizing NPP data with MOD17A3HGF yearly synthetic products. The data at a resolution of 1 km were resampled after pre-processing, including reprojection, Mosaic, and clipping, for input into the CASA model and data comparison.

#### 2.2.2 Meteorological data

The National Weather Service’s website (http://data.cma.cn) was utilized to download the meteorological data for this article, which included yearly precipitation and the percentage of average annual bright sunlight for the years 2000 to 2020. AUSPLIN interpolation software was used to analyze the data. After interpolation, the spatial resolution is 1 km when combined with the digital elevation model in the study area.

### 2.3 Methods

#### 2.3.1 NPP

In the CASA model, which is one of the several major light energy utilization models currently absorbed, vegetation net primary productivity was determined by variables absorbed by vegetation in photosynthetic active radiation (APAR) and in light energy conversion (ε). The NPP calculation model adopted in this study is an improved CASA model (Xue et al., 2022). Its calculation formula is as follows:


(1) NPP(x,t)=APAR(x,t)×ϵ(x,t)

In this equation, APAR (x, t) represents solar Photoactive radiation received by pixel *x* in month t, and in this case, ε (x, t) represents actual light energy utilization of pixel *x* in month *t*.

#### 2.3.2 SPEI

By factoring in the effects of precipitation and temperature on water balance, SPEI is used to evaluate and predict drought conditions at different time intervals. In this study, the monthly difference between evapotranspiration and precipitation is calculated after the potential evapotranspiration has been established using the Thornthwaite method. SPEI-1, SPEI-3, SPEI-6, and SPEI-12 are calculated for monthly, 3-month, 6-month, and 12-month intervals, respectively, after the difference series has been normalized using the 3-parameter log-logistic probability distribution. Methods for determining SPEI are described in detail in the cited works (Stagge et al., 2015; Wang et al., 2015).

The current research aims to investigate the effects of droughts on plant life. So, we’re looking at the drought condition that typically occurs during plant growth (April–September). Given that the drought condition is represented by the September SPEI-6 value for six months spanning the period from April to September, thus encompassing the entire growing season, the September SPEI-6 value has been used to formulate the drought loss rate curve.

#### 2.3.3 Correlation analysis

Analysis of correlation is a method that can be used to determine whether or not there is a connection between two or more factor variables. This can be done by comparing the levels of similarity between the factors. The Pearson correlation coefficient method has been selected to analyze the internal relationship between the two variables (Li et al., 2021). The formula used in this calculation is articulated below.


(2) $$R=\frac{\sum_{i=0}^{n}\left(x_{i}-\bar{x})(y-\bar{y}\right)}{\sqrt{\sum_{i=0}^{n}{\left(x_{i}-\bar{x}\right)}^{2}}\sqrt{\sum_{i=0}^{n}{\left(y-\bar{y}\right)}^{2}}}$$

In this equation, *x_i_* said the first years of NPP values, $\bar{x}$ Represents the mean value of NPP over the years, $\bar{y}$ Represents the mean SPEI over the years.

#### 2.3.4 Construction of drought loss rate curve

Based on CASA model, the regional NPP was estimated. As a representation of drought vulnerability, the drought loss rate curve based on CASA model was constructed. The details are as follows:

(1) Identification of drought. The SPEI index of vegetation growth season was used to judge the drought degree from April to September, and the SPEI values of all stations were no drought (SPEI≥0) or no more than 3 stations were no more than light drought (-1≤SPEI <0) month is drought free month. The identification results are shown in **Table 1**.
(2) According to the SPEI values identified at each station, the SPEI index raster map year by year, and the spatial resolution was consistent with the regional NPP raster map, both being 1km×1km. At the same time, GIS spatial analysis technology was used to extract the pixel value of SPEI index into the grid where the vegetation was located, and it was used to represent the drought risk value of the vegetation on a grid basis.
(3) Obtaining monthly NPP data for the study area for the 2000-2000, and the raster map of the regional vegetation growth season from April to September was selected from it, which was also extracted into the grid where the vegetation was located, and used as the grid value of vegetation drought vulnerability year by year, month by month.
(4) Calculate the drought loss rate, that is, the NPP loss rate of vegetation caused by drought. The NPP values of vegetation corresponding to normal months are considered as normal values, and the average values of vegetation NPP in all normal years are taken as the normal values of vegetation NPP in that month without drought. Therefore, the pixel-by-pixel drought loss rate can be calculated as follows:

**Table 1:** Monthly drought in the growing season at the Yinshanbeilu from 2000 to 2020.

Month	Normal year	Dry year
4	2000,2001,2002,2003,2005,2007,2010,2011,2012,2013,2015,2017,2019,2020	2004,2006,2008,2009,2014,2016,2018
5	2000,2002,2003,2005,2006,2007,2010,2011,2012,2014,2015,2016,2017,2019,2020	2001,2004,2008,2009,2013,2018
6	2000,2002,2003,2004,2006,2008,2010,2012,2013,2014,2015,2016,2017,2019	2001,2005,2007,2009,2011,2018,2020
7	2001,2002,2003,2004,2006,2008,2012,2013,2014,2015,2016,2019	2000,2005,2007,2009,2010,2011,2017,2018,2020
8	2000,2002,2003,2004,2008,2012,2015,2016,2018,2019,2020	2001,2005,2006,2007,2009,2010,2011,2013,2014,2017
9	2002,2003,2004,2008,2010,2012,2013,2014,2015,2016,2018,2019,2020	2000,2001,2005,2006,2007,2009,2011,2017


(3) $$NPP_{LDR}=\frac{NPP_{NO}-NPP_{DR}}{NPP_{NO}}\times 100\%$$

Where, NPP_LDR_ represents the NPP loss rate of drought-induced vegetation; NPP_NO_ represents the vegetation NPP in normal months; NPP_DR_ indicates the value of the NPP produced by plants during the month of drought.

It is acceptable that the loss rate of ecosystem is between 10%-20% (van Minnen et al., 2002). In this study, 20% NPP loss rate of vegetation is represented as the threshold line of drought loss. When the NPP loss rate of vegetation exceeds 20%, drought loss event begins to occur.

## 3 Results

### 3.1 Model accuracy verification

**Figure 2:**
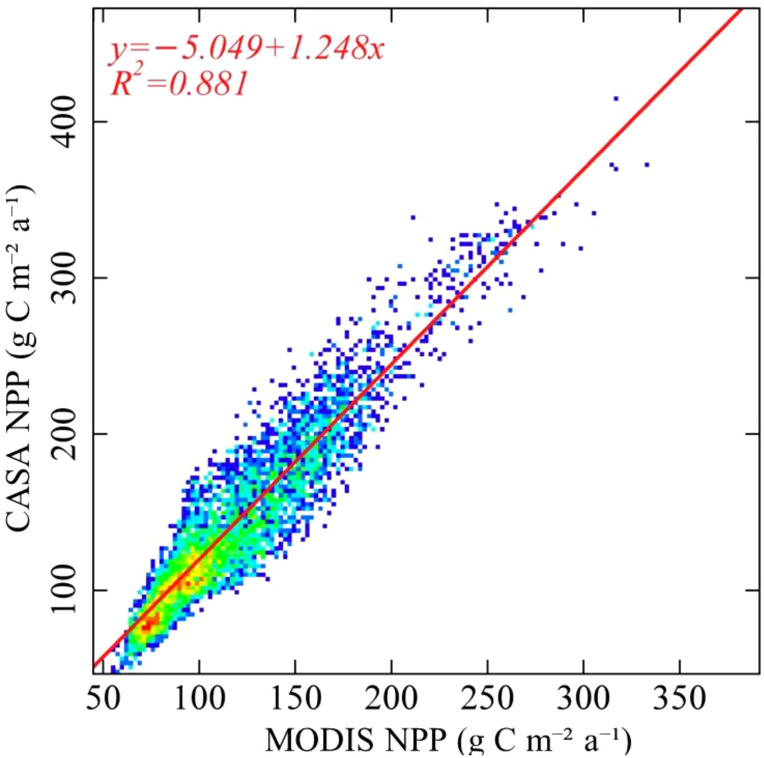
Accuracy verification of simulation results.

This study aimed to compare the estimated results obtained from the CASA model with the MOD17A3 data products spanning the period from 2000 to 2020, The two results are in good agreement(R^2^ = 0.881), thereby making it a suitable candidate for further analysis (**Figure 2**).

### 3.2 Spatial and temporal trends of SPEI

**Figure 3:**
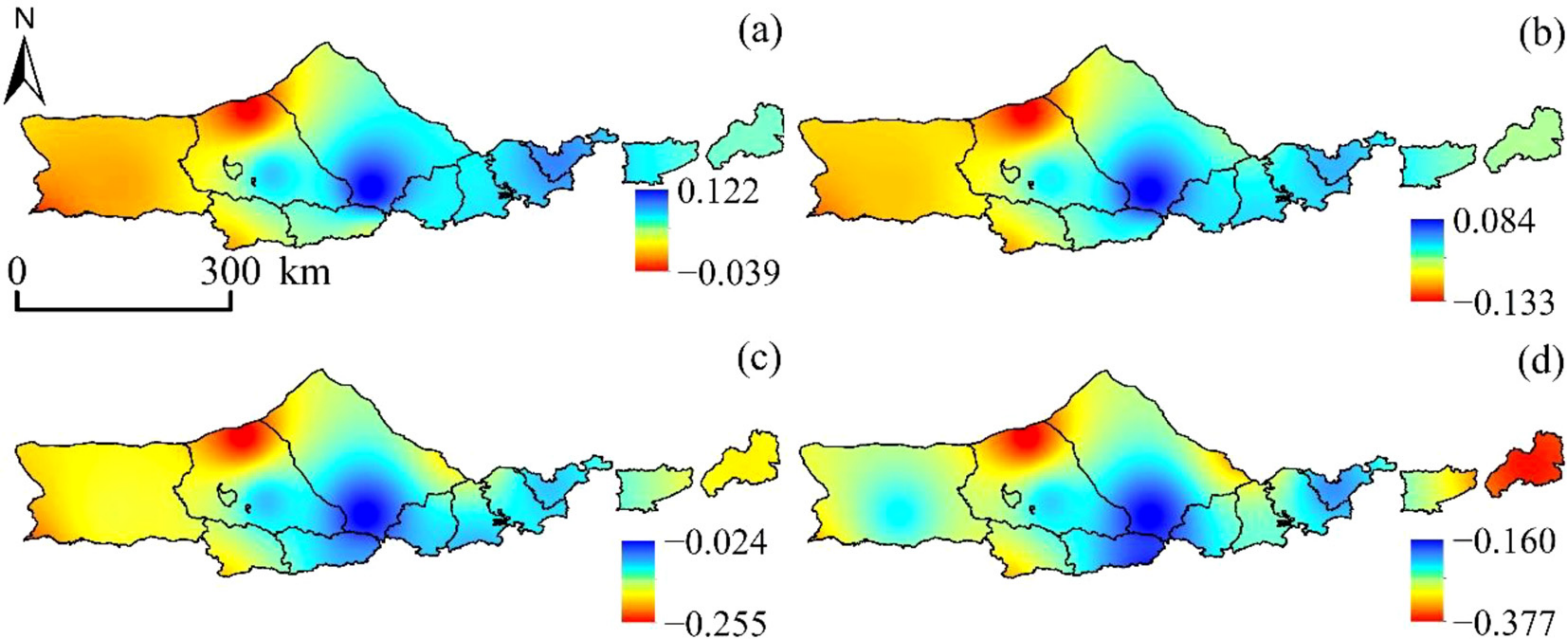
SPEI spatial distribution. **(A)** SPEI-1, **(B)** SPEI-3, **(C)** SPEI-6, and **(D)** SPEI-12.

Observations presented in **Figure 3** demonstrate that the SPEI displays varying degrees of accuracy in detecting drought conditions at the northern base of Yinshanbeilu depending on the time scale under consideration. Despite this, SPEI exhibits similar spatial distribution trends for drought at different scales, characterized by an east-west distribution pattern, depicting wet conditions in the east and dry conditions in the west.

**Figure 4:**
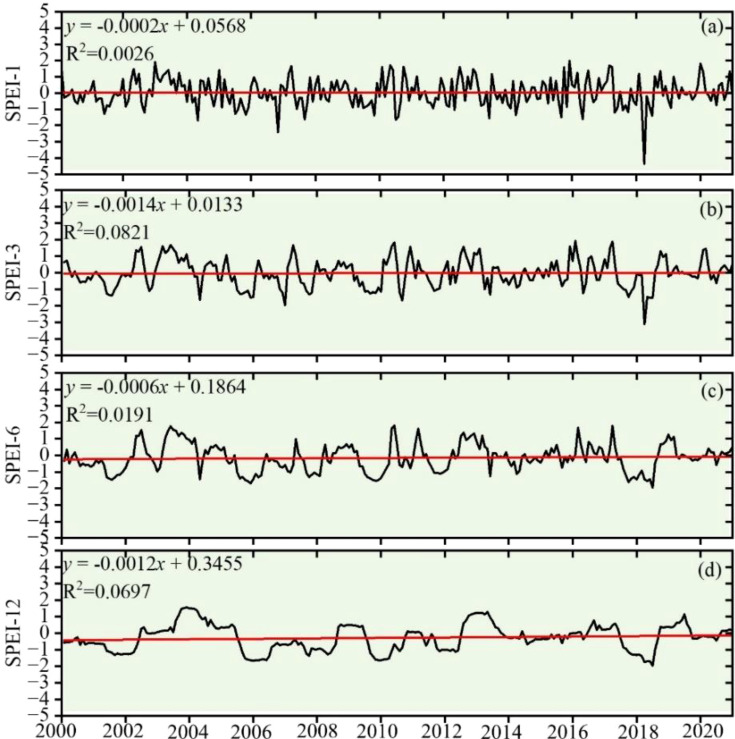
SPEI time variation trend. **(A)** SPEI-1, **(B)** SPEI-3, **(C)** SPEI-6, and **(D)** SPEI-12.

As can be seen in **Figure 4**, the sensitivity of the fluctuation of SPEI values at multiple scales from 2000 to 2020 is obviously different, showing a trend that is slightly decreasing at different time scales. This change can be seen across the board. The occurrence of drought is unpredictable and happens on a regular basis, and the multi-scale SPEI indexes in 2005, 2009, 2010, and 2019 show a clear turnaround, demonstrating a “down-rising-declining-rising trend.” The frequent occurrence of droughts followed by floods in each month is reflected by the fact that SPEI-1 experiences significant swings along the value of 0. This demonstrates the seasonal change pattern of dryness and wetness in the study area. The variability of the SPEI-12 is manageable, which enables it to comprehend the overarching pattern of drought progression.

**Figure 5:**
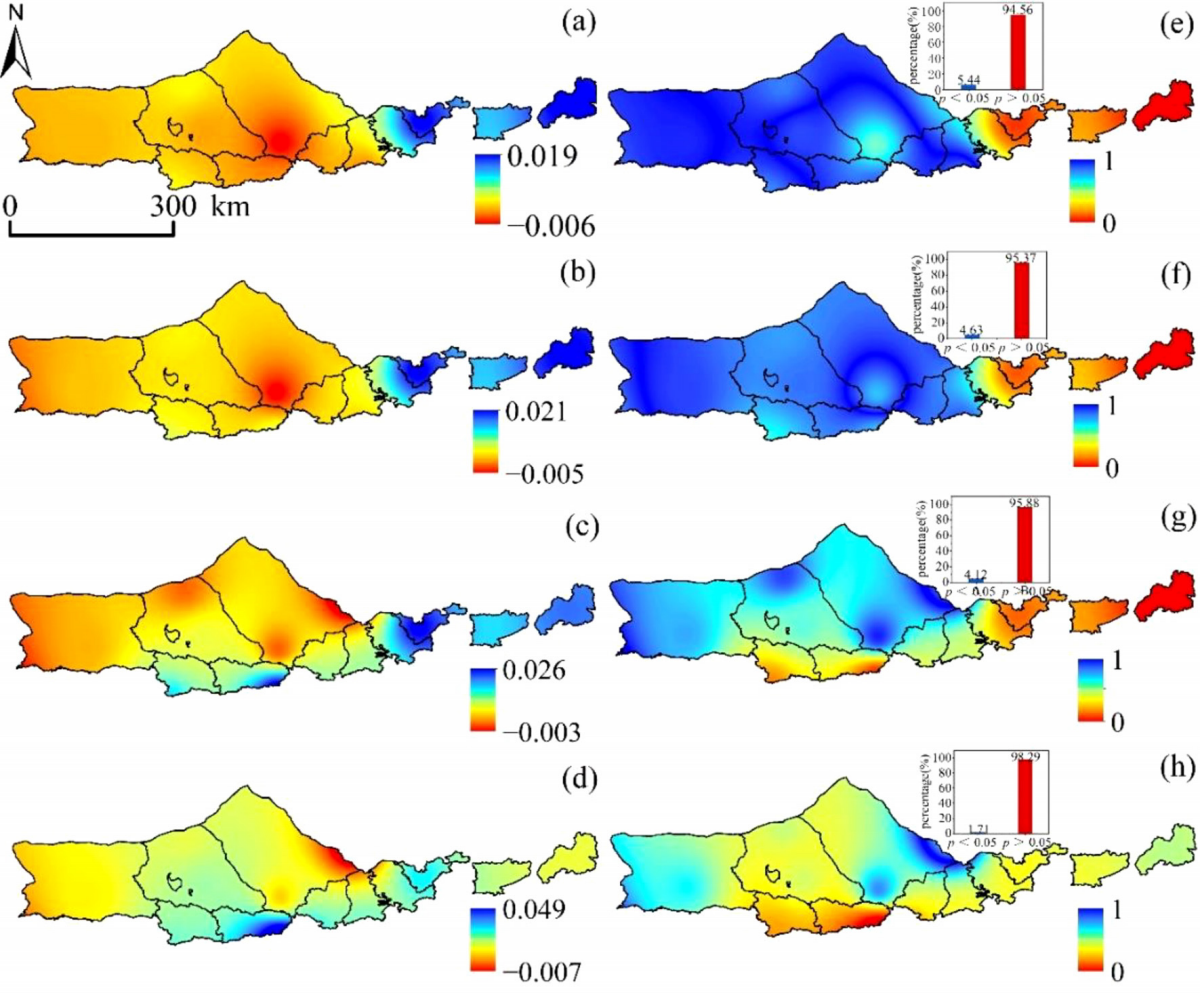
SPEI spatial variation trend:**(A)** SPEI-1, **(B)** SPEI-3, **(C)** SPEI-6, and **(D)** SPEI-12. SPEI significant change trend:**(E)** SPEI-1, **(F)** SPEI-3, **(G)** SPEI-6, and **(H)** SPEI-12.

**Figure 5** reveals that the variation trend of SPEI-1 is gradually increasing from west to east, with clear evidence of horizontal zonality rule. This can be seen to be the case with a value of -0.006~0.019/a. The area demonstrates a moderately weak downward trend, with the downward trend accounting for 84.25% of the total area and the upward trend accounting for 15.75% of the total area, respectively. Furthermore, only 5.44% of the area passes the significance test at the 0.05 level. They are found most frequently in the area of the study that is located to the east. Only 4.63% of the area met the criteria for significance when the test was performed at the level of 0.05, and the variation trend of SPEI-3 was -0.005~0.021/a. The SPEI-6 displayed a trend of -0.003~0.026/a, and the significance test found that only 4.12% of the area met the criteria for passing at the level of 0.05. The significance level of the significance test was set at 0.05, and the variation trend of SPEI-12 was -0.007~0.049/a. Only 1.71% of the area passed the significance test. In conclusion, between the years 2000 and 2020, the SPEI index in the western area exhibited a negative tendency across several time intervals, which suggested that the region tended to be dry. This pattern was consistent across all time scales. On the other hand, the SPEI index in the majority of the eastern area showed an upward trend, which suggested that these places exhibited a tendency towards being wetter. This was the case despite the fact that the western region continued to show a downward trend.

### 3.3 Spatial and temporal trends of NPP

**Figure 6:**
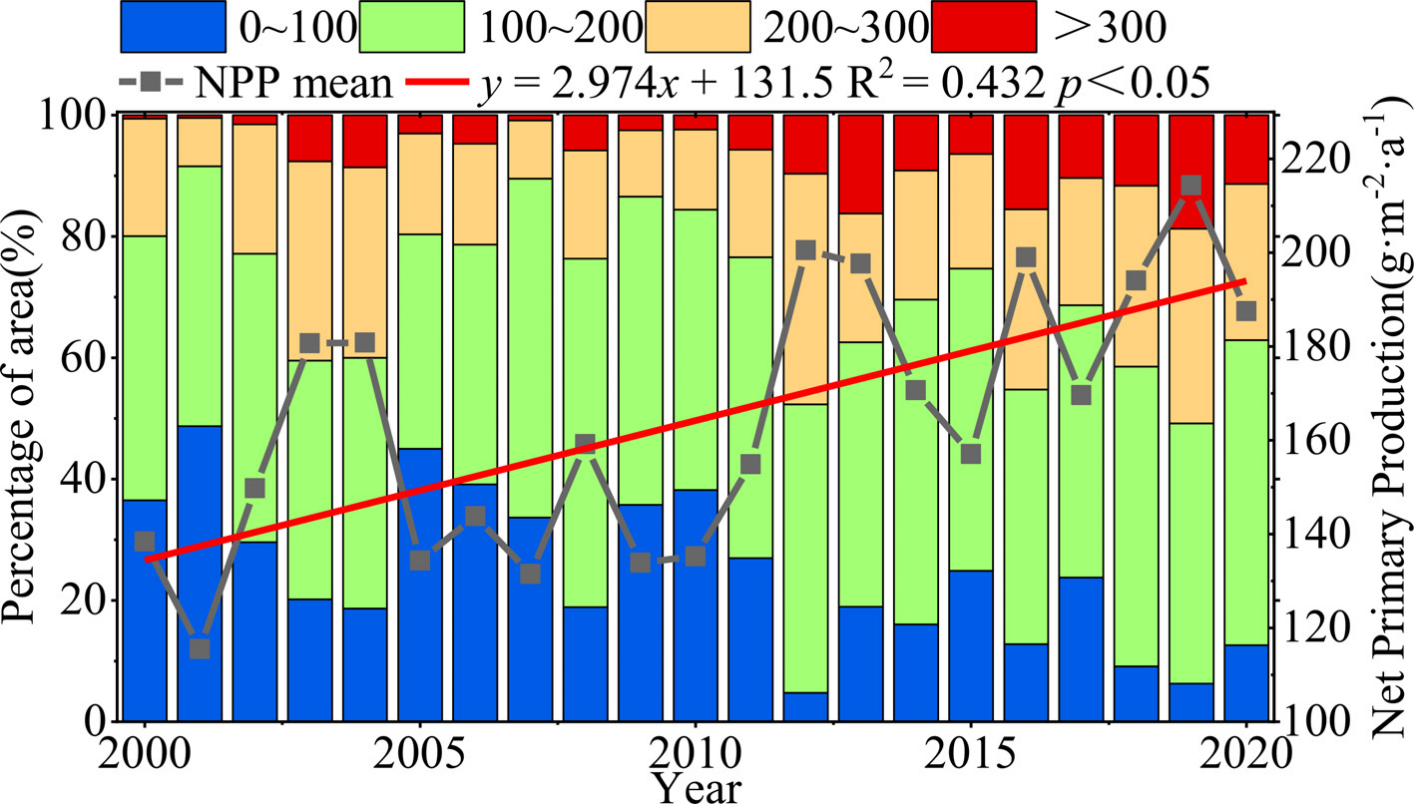
Interannual variation in vegetation NPP and variation in area occupied by NPP mean grading.

From 2000 to 2020, the mean NPP of vegetation at the northern foot of Yinshan showed great inter-annual variation, and showed a trend of fluctuation and increase, with an increase rate of 2.974g·m^-2^·a^-1^. The area percentage of NPP in the northern foothill of Yinshan was divided into four grades: 0∼100 g·m^-2^·a^-1^,100∼200 g·m^-2^·a^-1^,200∼300 g·m^-2^·a^-1^,>300 g·m^-2^·a^-1^. The results showed that the area proportion of vegetation NPP in the range of 0 ~ 100 g·m^-2^·a^-1^ was 45.01% in 2005, and the area proportion in this area tended to decrease during the whole study period. The NPP of vegetation ranges from 100 to 200 g·m^-2^·a^-1^, and the maximum area proportion is 35.29% ~ 57.39%. The mean NPP of vegetation in the area larger than 300 g·m^-2^·a^-1^ fluctuated between 0.46% and 16.22%, and showed a slow rising trend (**Figure 6**).

**Figure 7:**
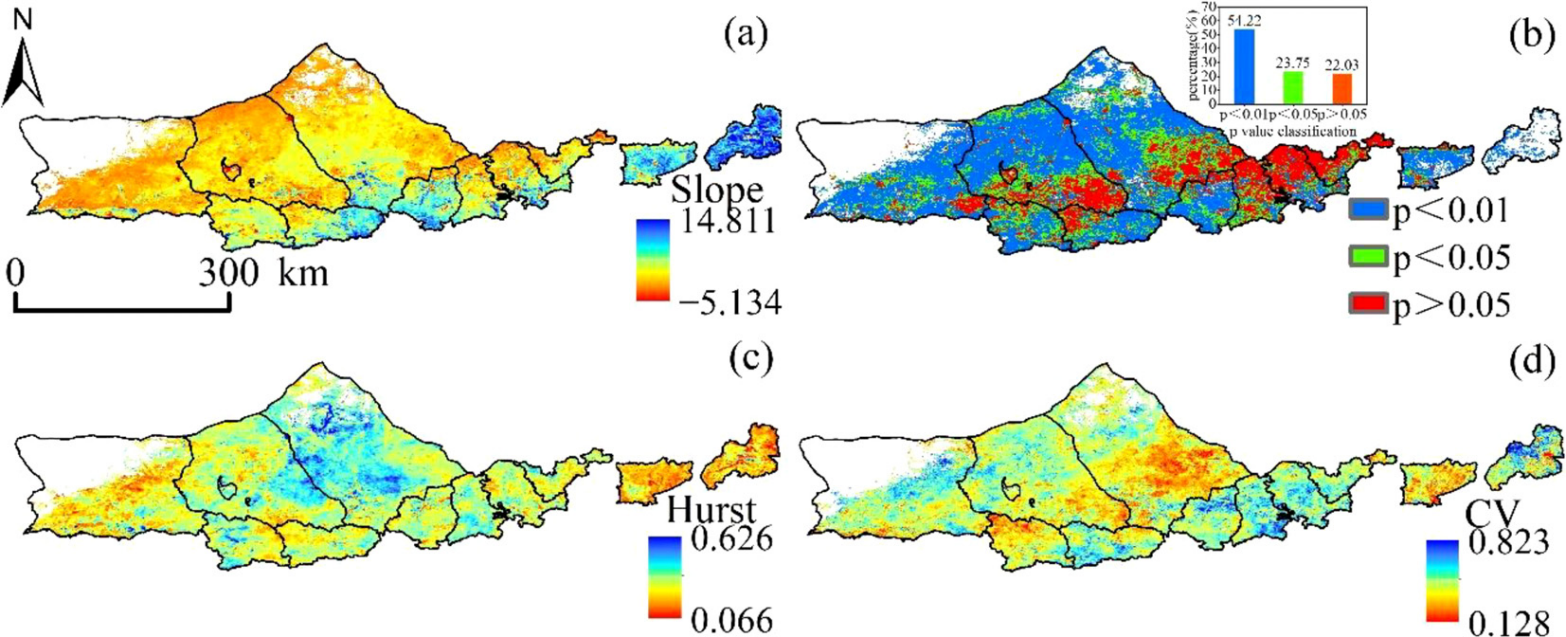
Yinshanbeilu change distribution features **(A)** spatial trend distribution, **(B)** significance distribution, **(C)** Hurst distribution, and **(D)** coefficient of variation distribution during 2000-2020.

The slope of the vegetation NPP fitted in the Yinshanbeilu from 2000 to 2020 varied from -5.135 to 14.811 g·m^-2^·a^-1^ (**Figure 7**), and the proportion of the region with a rising trend was 99.49%, spread in most parts of the research area. In Huade and Shangdu counties, as well as in the southwestern section of Siziwangqi, the majority of the land that showed no significant increase in the amount of vegetation NPP was located in the regions that were dominated by arable land. This accounted for 22.03% of the total land area (*p*> 0.05). The significance test (*p*> 0.05) was passed by the regions that accounted for 23.75% of the total area, while the highly significant test ((*p <*0.01) was passed by the regions that accounted for 54.22% of the total area. The Hurst index of vegetation NPP in the Yinshanbeilu ranged from 0.066 to 0.626, with a mean value of 0.396, and the proportion of the image elements with a Hurst index less than 0.5 was 62.36%. This suggests that the inverse persistence of vegetation NPP change in the Yinshanbeilu is greater than the positive persistence in the future, which implies that the tendency of vegetation NPP shift will be reverted at some point in the future. The yearly mean value of the NPP produced by vegetation in the northern half of the Yinshanbeilu is predicted to have a low coefficient of variation from the years 2000 to 2020, and the percentage of the regional image elements with the coefficient of variation below 0.25 is 89.89%, which indicates that the mean value of the vegetation NPP time series in the northern part of the Yinshanbeilu is relatively stable. This was determined by comparing the percentage of the regional image elements with the coefficient of variation below 0.25. The coefficients of variation ranging from 0.250 to 0.823 are most often seen in regions that have experienced significant shifts in land use and regions that have been significantly impacted by human activities.

### 3.4 The correlation between SPEI and NPP

**Figure 8:**
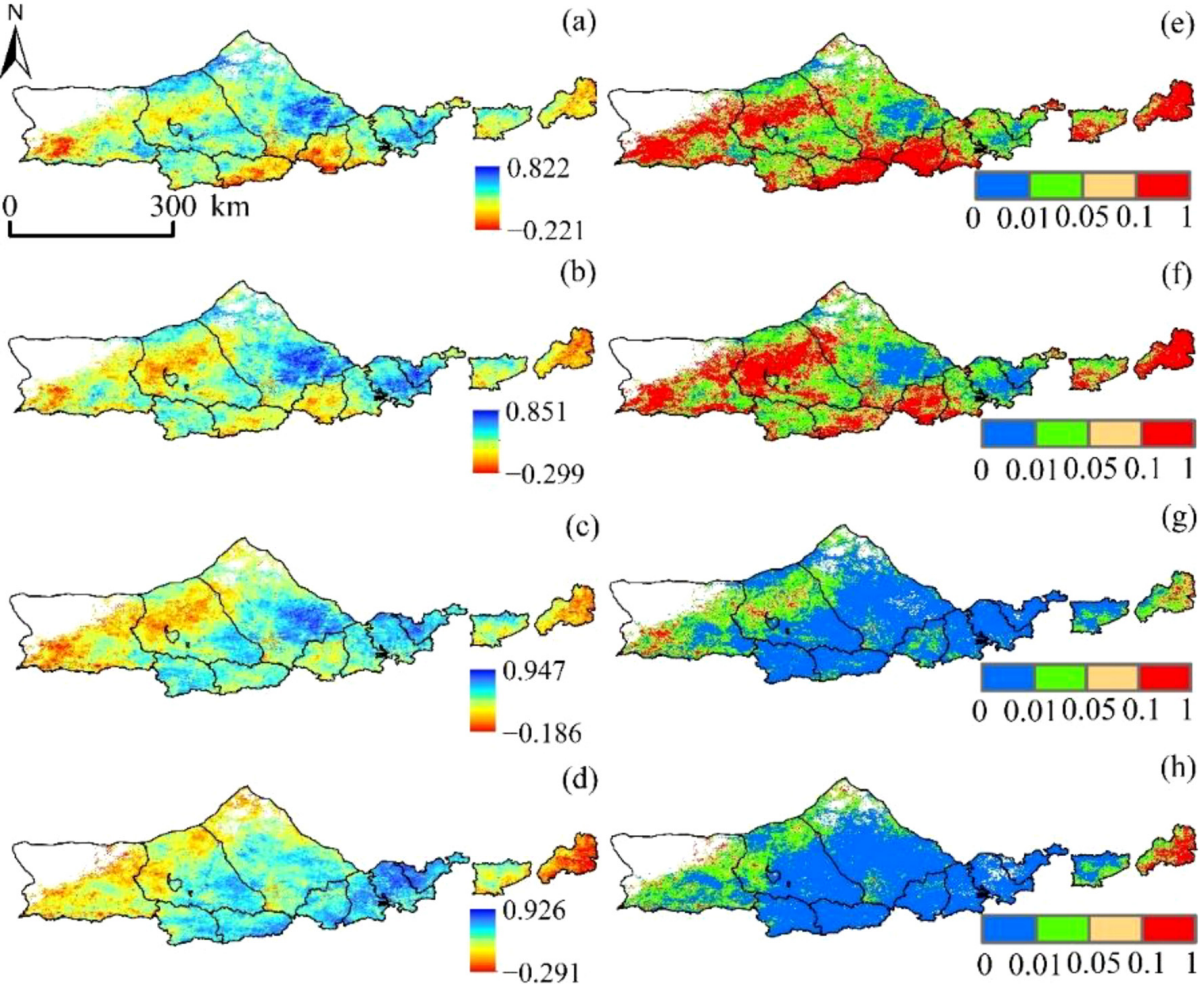
Correlation between SPEI and vegetation NPP at different time scales:**(A)** SPEI-1, **(B)** SPEI-3, **(C)** SPEI-6, and **(D)** SPEI-12. The significance of SPEI and vegetation NPP at different time scales: **(E)** SPEI-1, **(F)** SPEI-3, **(G)** SPEI-6, and **(H)** SPEI -12.

SPEI (SPEI -1, SPEI -3, SPEI -6, and SPEI -12) and vegetation NPP had correlation values of -0.221 to 0.822, -0.299 to 0.851, -0.186 to 0.947, and -0.291 to 0.926, respectively, over a variety of time periods (**Figure 8**). The average coefficients of correlation were 0.408, 0.427, 0.602, and 0.623. The overall geographical distribution is “low in the southwest and high in the northeast.” The percentages of SPEI positively linked with NPP at each time scale were 75.89%, 77.23%, 81.35%, and 83.56%, with 42.53%, 48.15%, 90.72%, and 92.75%, respectively, passing the significance test (*p*<0.05). vegetation NPP and SPEI that strengthened with decreasing SPEI time scale, indicating that Yinshanbeilu’s NPP responded relatively well to drought changes on an annual scale but poorly to those on a medium- and short-term basis, especially to a short-term surface water anomaly and a seasonal scale.

### 3.5 Loss rate of NPP under drought stress

**Figure 9:**
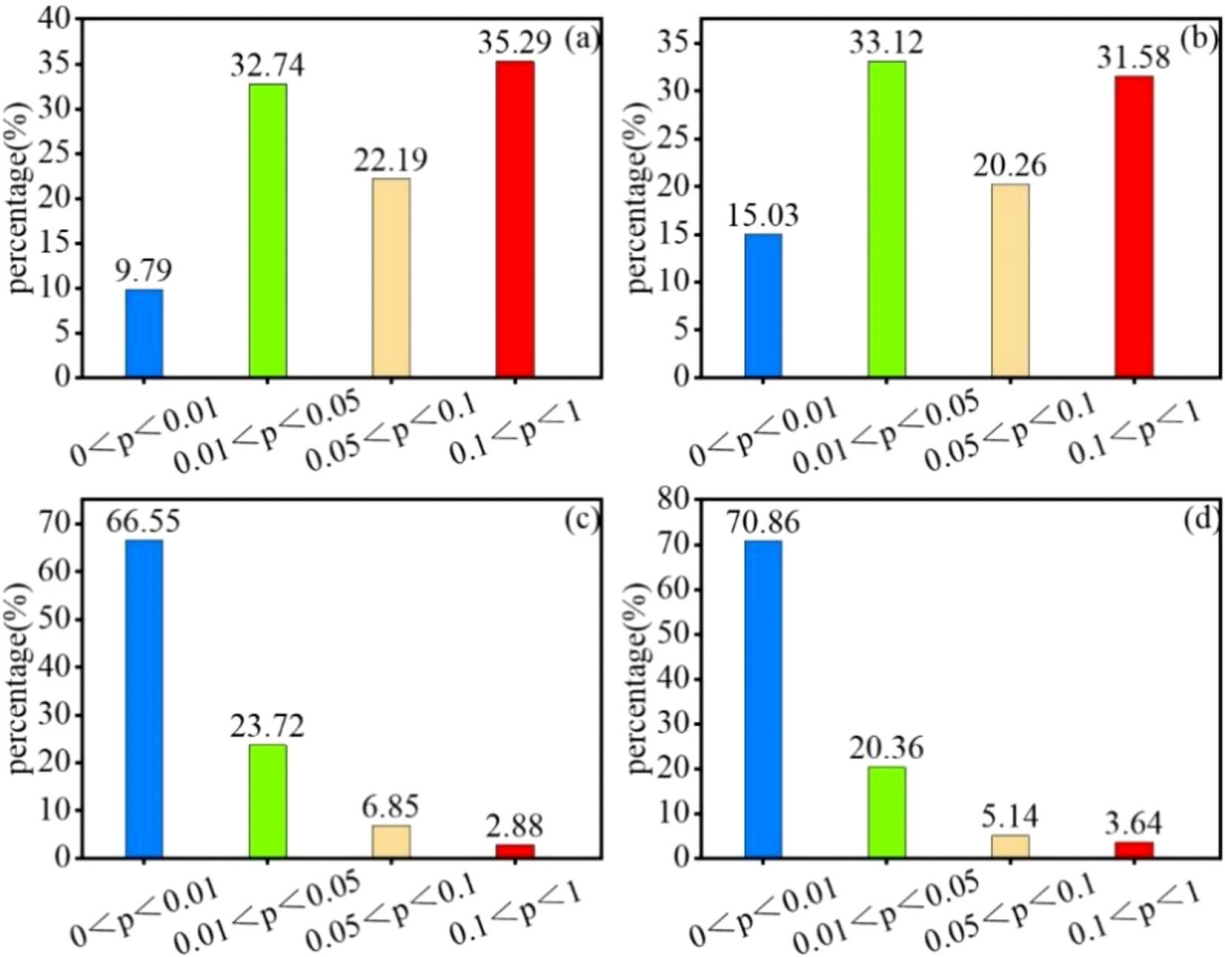
Percentage significance of SPEI vs. NPP at different time scales. **(A)** SPEI-1, **(B)** SPEI-3, **(C)** SPEI-6, and **(D)** SPEI-12.

As can be seen from **Figure 9**, The variation range of NPP loss rate caused by drought was 2.351~46.238%, 13.621~49.596%, 0.849~39.873%, 4.023~49.921% and 0.397-47.685 in different growth periods of vegetation in the vegetation growing season (each month was taken as a growth period of vegetation in this study) %, 0.558~49.886%, and the average loss rates were 23.64%, 28.53%, 29.18%, 32.86%, and 32.95%, respectively. Generally, the spatial distribution pattern is “low in the west and high in the east”.

**Figure 10:**
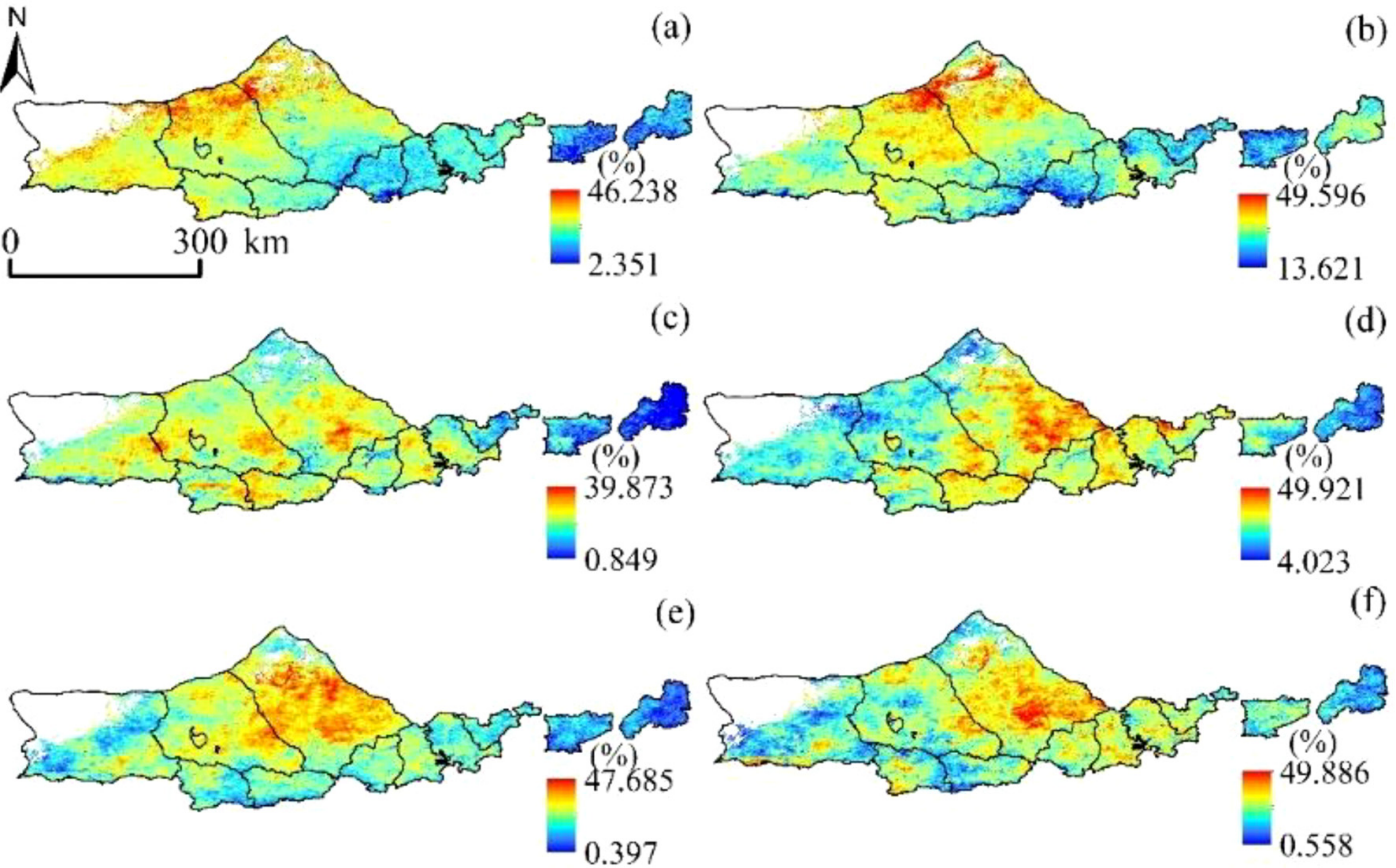
NPP loss rate of vegetation due to drought occurrence. **(A)** April, **(B)** May, **(C)** June, **(D)** July **(E)** August, and **(F)** September.

In this study, positive pixels with SPEI index were excluded and NPP losses of vegetation were only considered when the SPEI index represented drought (**Figure 10**). Then, NPP loss rates caused by different drought degrees were fitted to obtain monthly drought loss rate curves of regional vegetation growth seasons. The results passed the 0.05 significance test. The R^2^ values of each growth period were 0.81, 0.81, 0.82, 0.89, 0.84 and 0.85, respectively, indicating that the curve fitting accuracy of drought loss rate was high, which could be used for drought loss analysis.

**Figure 11:**
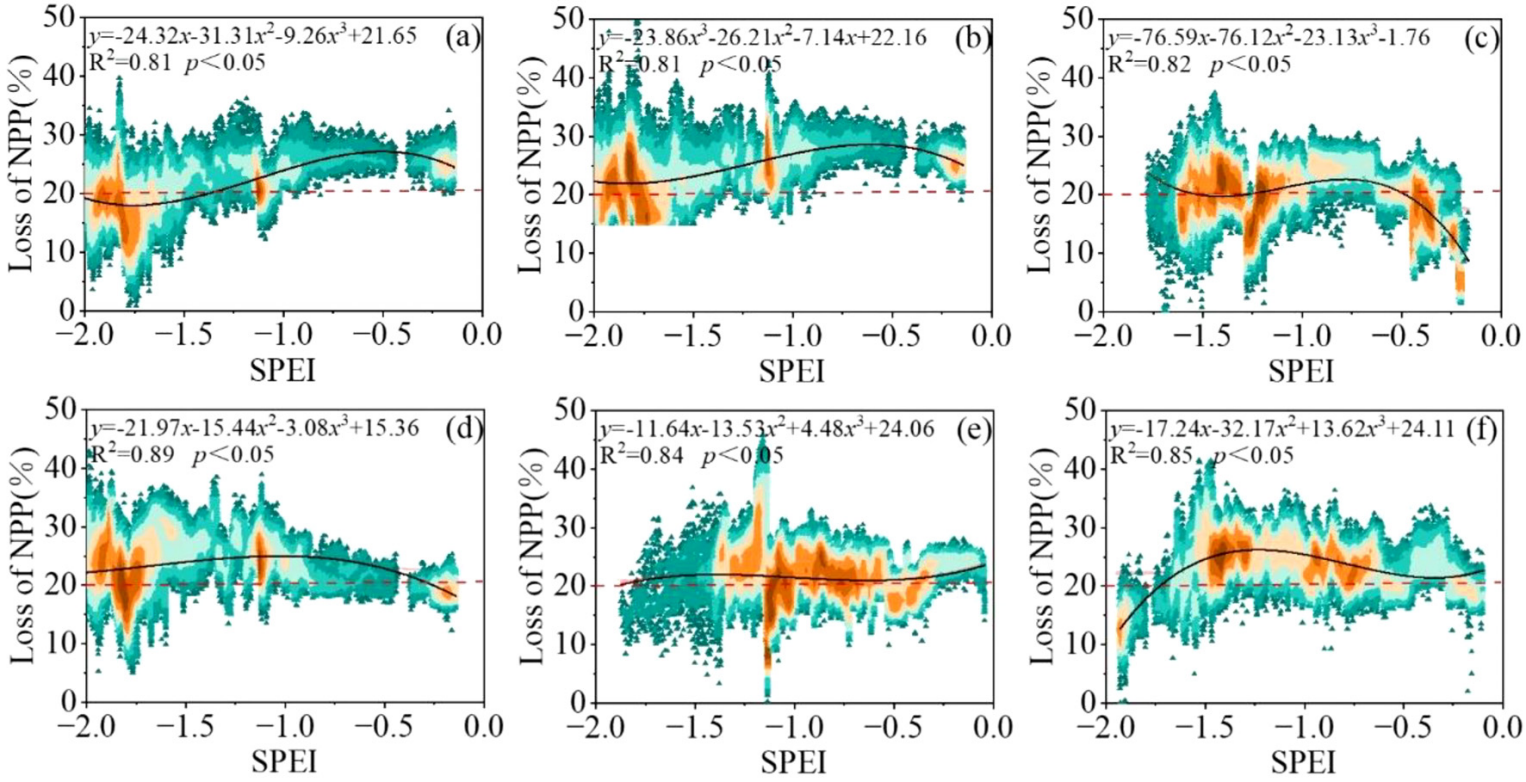
Drought loss rate curve of different months. **(A)** April, **(B)** May, **(C)** June, **(D)** July **(E)** August, and **(F)** September. (Note:The red dotted line represents the threshold for drought damage).

On the whole, vegetation NPP is more sensitive to the occurrence and development of drought. With the increase of drought degree, the loss rate of vegetation NPP in each growth period also fluctuates and increases gradually, and the fluctuation range of the loss rate is 20-50% (**Figure 11**). In the drought loss rate curve in April, there were two obvious peaks at SPEI of -1.25 and -1.75. In April, the grassland is in the greening period. When the drought degree is light drought, the NPP loss rate of vegetation fluctuates between 20-35%, and the fluctuation is relatively gentle. When the drought degree changed from light drought to severe drought, the NPP loss rate increased rapidly and fluctuated between 35 and 45%. When the degree of drought is extreme drought, the NPP loss rate of vegetation changes slowly and stabilizes between 45-50%, which may be due to the fact that the soil has a certain moisture in the early stage, so that the NPP of vegetation can still maintain at about 50% even if the extreme drought disaster occurs. From May to August, with the worsening of drought, the loss rate showed an increasing trend. Among them, the NPP loss rate fluctuated between 20-50% in May. When the drought degree reached extreme drought, the loss stabilized at 45% and fluctuated with the severity of drought again. In July, due to the low drought degree in July from 2000 to 2020, the maximum drought level only reached the severe drought level, and the NPP loss of vegetation showed an exponential rising trend. However, despite the low drought degree, the NPP loss of vegetation fluctuated between 20-40%, indicating that the drought occurred in July had a greater impact on grassland NPP. This month is the most critical month for the accumulation of forage production. From June to August, when the degree of drought reached severe drought or extreme drought, the increase rate of NPP loss rate was slow and fluctuated between 40-50%. For September, the NPP loss rate of vegetation also showed an increasing trend. When the drought degree reached between moderate drought and severe drought, the NPP loss rate fluctuated between 40-45%. However, when the drought degree reached severe drought, the NPP loss rate showed an accelerating trend, which may be related to the fact that grassland harvesting and storage had begun in some areas in September. In conclusion, NPP of vegetation is sensitive to drought. Even under light drought conditions, NPP of grassland will lose 10-20%, especially in July.

## 4 Discussion

### 4.1 Characteristics of vegetation NPP and drought

In this study, it was found that NPP of vegetation at the Yinshanbeilu increased significantly at a rate of 2.974 g·m^-2^·a^-1^ from 2000 to 2020 (*p* < 0.05), which is basically consistent with the results of (Liu et al., 2020b). However, there are still uncertainties about the main driving factors of this change. Zhao et al. (2019b) the accumulation of organic matter in vegetation was jointly influenced by increased precipitation and temperature, with precipitation having a stronger impact than temperature. Liu et al. (2021a) pointed out the surface temperature has a considerable effect on the development of plants, although the degree to which this influence is exerted changes with height. Human activities have a bigger influence on the growth of vegetation in low-altitude locations than temperature does; nevertheless, warming has a more dramatic effect on boosting the productivity of vegetation in high-altitude places. The majority of the study area is made up of agricultural land, low-cover grassland, and desert. The area suffers from an unreliable water supply, low precipitation, high evaporation, low soil water content, and severe drought, all of which inhibit the accumulation and fixation of carbon in the region’s plant life (Zhang et al., 2020). Concurrently, human activities demonstrate a dualistic influence on the environment. Furthermore, the presence of radiation, atmospheric concentration of CO_2_, crop yield, and their compounded consequences can also generate a discernible impact on the escalation of vegetation productivity (Guo et al., 2021; Pei et al., 2020; Yang et al., 2020).

In this research, the SPEI index was calculated by using the temperature and precipitation data collected from a total of 12 sites. It was discovered that the consequences of climate change led to a less severe drought in the eastern areas, while the circumstances of drought became worse in the western regions. This was the outcome of a drop in the severity of drought in the eastern regions. The western portions of the research area were mostly home to the regions that were experiencing the most severe levels of drought. The steady ascent in amounts of precipitation was the key contributor to this development, and it was accountable for it. Concurrently, a decrease in wind speed and an increase in the sunlight duration greatly contributed to the reduction in potential evapotranspiration, which is consistent with the results of the research that used the SPEI index (Pei et al., 2020; Wei et al., 2022b). This indicates that SPEI can accurately reflect drought conditions in the study area.

### 4.2 Effects of drought on vegetation NPP

Atmospheric drought caused by insufficient precipitation will affect soil, vegetation, runoff and groundwater to varying degrees, and then cause agricultural drought and ecosystem drought (Lai et al., 2018). Drought-induced water deficit and osmotic stress can significantly impede plant growth and development, reduce crop yields, and may even result in plant mortality under severe cases (Doughty et al., 2015). Numerous researchers from the United States have investigated the effect that drought has on vegetation response at regional scales. The findings of these studies, which were conducted in areas such as the Loess Plateau, Inner Mongolia, Northeast China, and Northwest China, validate the substantial ramifications that drought has on the productivity of vegetation (Feng et al., 2016; Guo et al., 2021; Yan et al., 2021; Zhou et al., 2015). Within the scope of this specific research, an investigation into the degree to which the levels of NPP and SPEI at Yinshanbeilu changed throughout the course of a variety of time periods was carried out and analyzed. The findings showed that there is a positive link between the values of NPP and SPEI, which suggests that a water shortage may have a major influence on the development of vegetation. In addition, when looking at the regions that were subjected to the significance test, it was discovered that the correlation between drought and NPP increased with the accumulation of drought over a more extended time scale. This finding highlights the greater impact that long-term drought has on the NPP of vegetation in Yinshanbeilu. These results are consistent with those of other research that shown the impact of drought accumulation on NDVI in geographical areas that are characterized as being generally dry (Li et al., 2020b). Varied levels of water availability may either facilitate or impede the progress of plant growth and maturation, thereby influencing the ongoing phase of the plant’s life cycle. In essence, the current state of a plant’s life cycle is contingent upon the preceding stage (Lei et al., 2015). Moreover, it is important to note that rising temperatures and extreme weather events, including drought, have a direct impact on the evapotranspiration of both soil water and vegetation canopy water. This results in heightened levels of transpiration and surface dryness, ultimately causing erosive damage by strong solar radiation and wind, along with extreme precipitation. Such erosive effects lead to decreased grip of vegetation roots, further compounding the increase of evapotranspiration in deep soil (Yan et al., 2022).

Yinshanbeilu finds itself situated in areas defined as arid and semi-arid, facing the persistent challenges of wind and water erosion throughout the year. Drought conditions may impede vegetation growth, reduce biomass, and elevate vegetation mortality rates, ultimately exacerbating soil desertification. Some areas have surprisingly yielded a negative correlation between drought and NPP. This discovery is suggested to arise from the plant species in these arid and semi-arid areas, who have adapted to their harsh environment with notable drought tolerance, therefore exhibiting exceptional growth patterns even under drought stress. Additionally, such vegetation can lower their water stress by way of reduced stomatal conductance, which enhances water use efficiency, and augments vegetation growth as a result (Fathi-Taperasht et al., 2022; Qin et al., 2022).

### 4.3 Uncertainties and limitations

In the current investigation, we will be computing the standard precipitation evapotranspiration index by making use of data that has been interpolated from several meteorological stations. After that, we evaluate the geographical as well as the temporal patterns of drought, keeping in mind that there is a possibility that there will be more ambiguity in the findings of the study. It is possible to construct a more complex regional drought monitoring model through the combination of data from remote sensing and observations from ground-based meteorological stations as the accuracy of meteorological satellite data continues to increase in tandem with the extension of relevant data series over a longer period of time (Wu et al., 2022; Xing et al., 2023). This is something that can be done by integrating the data.

The application of the CASA model served as a means to simulate vegetation NPP in the Yinshanbeilu and compare it with the MODIS NPP data for validation. However, the validation and analysis of field measurement data in specific geographical areas require further attention. Conducting field monitoring and data collection will be prioritized in the future to enhance the demonstrable effectiveness of the CASA model in monitoring the NPP of vegetation in the Yinshanbeilu.

Current research investigating the determinants of vegetation NPP has predominantly concentrated on drought-related factors, with limited attention being paid to the effects of human activities. Such activities may significantly impact the distribution and decomposition of NPP, thereby having direct implications on an ecosystem’s material and energy cycles (Zhang et al., 2022). According to the findings of previous studies, human activities have two fundamental impacts on the NPP of plants. To begin, human activities may cause shifts in the kinds of land use, which may ultimately result in a decrease in the NPP of plants. Second, the intentional conservation efforts that have been performed by humans are the key contributors to the rise in the NPP of plants. Grassland ecosystems, in compared to other types of vegetation communities, are more sensitive to the effect that human activities have on the NPP of vegetation and are hence more fragile. As a result, the next step in study will concentrate on determining how changes in NPP occur in vegetation as a result of the effect of human activities.

## 5 Conclusions

The present study aimed to develop drought loss rate curves through the assessment of net primary productivity losses caused by varying intensities of drought. This approach can serve as a valuable tool for offering guidance on the sustainable development of terrestrial ecosystems in Yinshanbeilu, as well as mitigation measures for tackling drought-related disasters.

(1) During 2000-2020, the western multi-timescale SPEI indices all show a decreasing trend, indicating that the region tends to be arid, while most of the eastern regions show an increasing trend in multi-timescale SPEI indices, indicating that these regions show a trend of becoming wetter.
(2) The mean vegetation NPP values from 2000 to 2020 showed a large interannual variation and a fluctuating upward trend with a growth rate of 2.974 g·m^-2^·a^-1^.
(3) The proportion of places having a positive correlation between SPEI and vegetation NPP at various time scales was 75.89%, 77.23%, 81.35%, and 83.56%, respectively. This demonstrates that the connection between vegetation NPP and SPEI grew when the SPEI time scale was raised.
(4) Vegetation NPP is more sensitive to drought response, and even light drought conditions can lead to 10-20% loss of grassland NPP, especially in July when vegetation NPP is more sensitive to drought response.

## Data Availability

The raw data supporting the conclusions of this article will be made available by the authors, without undue reservation.
